# Predictive processing increases intelligibility of acoustically distorted speech: Behavioral and neural correlates

**DOI:** 10.1002/brb3.789

**Published:** 2017-08-04

**Authors:** Maria Hakonen, Patrick J. C. May, Iiro P. Jääskeläinen, Emma Jokinen, Mikko Sams, Hannu Tiitinen

**Affiliations:** ^1^ Brain and Mind Laboratory Department of Neuroscience and Biomedical Engineering (NBE) School of Science Aalto University Aalto Finland; ^2^ Department of Physiology Faculty of Medicine University of Helsinki Helsinki Finland; ^3^ Medical Research Council Institute of Hearing Research School of Medicine The University of Nottingham Nottingham UK; ^4^ Special Laboratory Non‐Invasive Brain Imaging Leibniz Institute for Neurobiology Magdeburg Germany; ^5^ Department of Signal Processing and Acoustics School of Electrical Engineering Aalto University Aalto Finland; ^6^ Finland

**Keywords:** acoustic distortion, comprehension, functional magnetic resonance imaging, intelligibility, memory, speech

## Abstract

**Introduction:**

We examined which brain areas are involved in the comprehension of acoustically distorted speech using an experimental paradigm where the same distorted sentence can be perceived at different levels of intelligibility. This change in intelligibility occurs via a single intervening presentation of the intact version of the sentence, and the effect lasts at least on the order of minutes. Since the acoustic structure of the distorted stimulus is kept fixed and only intelligibility is varied, this allows one to study brain activity related to speech comprehension specifically.

**Methods:**

In a functional magnetic resonance imaging (fMRI) experiment, a stimulus set contained a block of six distorted sentences. This was followed by the intact counterparts of the sentences, after which the sentences were presented in distorted form again. A total of 18 such sets were presented to 20 human subjects.

**Results:**

The blood oxygenation level dependent (BOLD)‐responses elicited by the distorted sentences which came after the disambiguating, intact sentences were contrasted with the responses to the sentences presented before disambiguation. This revealed increased activity in the bilateral frontal pole, the dorsal anterior cingulate/paracingulate cortex, and the right frontal operculum. Decreased BOLD responses were observed in the posterior insula, Heschl's gyrus, and the posterior superior temporal sulcus.

**Conclusions:**

The brain areas that showed BOLD‐enhancement for increased sentence comprehension have been associated with executive functions and with the mapping of incoming sensory information to representations stored in episodic memory. Thus, the comprehension of acoustically distorted speech may be associated with the engagement of memory‐related subsystems. Further, activity in the primary auditory cortex was modulated by prior experience, possibly in a predictive coding framework. Our results suggest that memory biases the perception of ambiguous sensory information toward interpretations that have the highest probability to be correct based on previous experience.

## 1 INTRODUCTION

Speech comprehension is driven by the acoustics of the speech signal and by memory representations (referred to as internal models, schemas, memory templates, or endograms in perception and memory research) that facilitate the interpretation of acoustic information by mediating predictive information from experience into the current perception. However, the brain areas and neural mechanisms involved in integrating these two forms of information are still largely unknown. One reason for this may be that speech comprehension has usually been studied by comparing brain responses to acoustically different stimuli, which makes it challenging to distinguish whether the changes in the brain responses reflect speech intelligibility or the acoustic structure of the stimulus.

In our recent magnetoencephalography (MEG) and behavioral studies (Hakonen et al., 2016; Tiitinen, Miettinen, Alku, & May, 2012), we introduced an experimental paradigm where an acoustically distorted sentence of low intelligibility becomes easier to understand after a *single* presentation of the intact version of the same sentence, even when these presentations are separated by several minutes. We found that this intelligibility enhancement is greater for full sentences than for individual words, and that distorted vowels remain unrecognizable. This paradigm allows one to record brain activity associated with different levels of speech intelligibility while keeping the stimulation fixed. As such, it resembles the procedures used in recent brain studies in which the intelligibility of acoustically distorted words or sentences was increased by presenting the disambiguating stimulus (in either a written or spoken form) at the same time or immediately after the distorted word or sentence (Clos et al., 2014; Hervais‐Adelman, Carlyon, Johnsrude, & Davis, 2012; Sohoglu & Davis, 2016; Sohoglu, Peelle, Carlyon, & Davis, 2012; Tuennerhoff & Noppeney, 2016; Wild, Davis, & Johnsrude, 2012a; Zekveld, Rudner, Johnsrude, Heslenfeld, & Rönnberg, 2012). Also, this immediate pairing of disambiguating stimuli with distorted ones has been used in the studies addressing perceptual learning where subjects become adept at deciphering noise‐vocoded speech (Davis, Johnsrude, Hervais‐Adelman, Taylor, & McGettigan, 2005; Giraud et al., 2004; Hervais‐Adelman et al., 2012). Our paradigm differs from these procedures in several important ways. First, rather than presenting the disambiguating stimulus immediately together with the distorted sound, the presentation of the intact speech sound occurs minutes before the presentation of the distorted sound. Second, none of the above paradigms (ours included) may be considered close, ecologically valid approximations of the conditions under which the brain learns to decipher noisy speech signals. However, one could argue that our paradigm has ecological merit, because disambiguating stimuli are seldom immediately available in real‐world situations. Further, our paradigm suggests that the processing of noisy speech is robust, requiring no perceptual training but, rather, involves the rapid recruitment of presentations of speech signals in long‐term memory. Third, despite the long delay between presentations, there is an intelligibility “pop‐out” effect in that the distorted word or sentence is easy to understand.

Intelligible speech is thought to be processed hierarchically in the human brain, with the primary auditory cortex reflecting acoustic differences in speech stimuli, and the temporal cortical regions anterior and posterior to the auditory cortex being sensitive to speech intelligibility and less sensitive to acoustic structure (for a review see Peelle, Johnsrude, & Davis, 2010). Recently, the hierarchical model of the processing of speech has been extended to include motor, premotor, prefrontal, and posterior inferiotemporal regions (Peelle et al., 2010). However, the strategies of the human brain to resolve semantic content of speech may differ under acoustically optimal and suboptimal conditions. Indeed, contradicting the hierarchical model of speech comprehension, activity in the primary auditory cortex has been shown to reflect speech intelligibility when speech is acoustically distorted (Wild et al., 2012b). Furthermore, speech comprehension specifically in acoustically adverse conditions has been associated with several brain areas including the left inferior frontal gyrus (Clos et al., 2014; Giraud et al., 2004; Hervais‐Adelman et al., 2012; Obleser & Kotz, 2010; Obleser, Wise, Dresner, & Scott, 2007; Shahin, Bishop, & Miller, 2009; Wild et al., 2012a), the anterior cingulate cortex (Erb, Henry, Eisner, & Obleser, 2012; Giraud et al., 2004), the anterior insula (Adank, 2012; Erb, Henry, Eisner, & Obleser, 2013; Giraud et al., 2004; Hervais‐Adelman et al., 2012; Shahin et al., 2009), the middle frontal gyrus (Giraud et al., 2004; Sohoglu et al., 2012), and the supplementary motor cortex (Adank, 2012; Erb et al., 2013; Hervais‐Adelman et al., 2012; Shahin et al., 2009). Subcortical brain structures may be involved in the adaptive plasticity that allows comprehension of even severely degraded speech (Guediche, Blumstein, Fiez, & Holt, 2014a; Guediche, Holt, Laurent, Lim, & Fiez, 2014b; Jääskeläinen et al., 2011). Together, these studies indicate that the current models of the comprehension of intact speech cannot fully describe the mechanisms of speech comprehension in acoustically suboptimal conditions.

Here, we used behavioral and fMRI experiments to study the neural mechanisms underlying the disambiguation of degraded spoken sentences in situations where the subject has previously been exposed to the intact versions of the sentences. In this experimental design, a degraded sentence is first experienced as very difficult to understand and, after a single presentation of its intact counterpart, the intelligibility of this same degraded sentence reaches near‐perfect level. This allows collecting behavioral and neural responses across varying intelligibility conditions while keeping the acoustic stimulation fixed. In contrast to previous studies that have disambiguated sentences of low initial intelligibility by pairing these with their written or acoustically intact auditory counterparts, we avoided such pairing by presenting a set of sentences three times: in the first presentation, the sentences were acoustically distorted; in the second presentation, the sentences were intact; in the final presentation, the acoustically distorted versions were presented again. In the behavioral experiment, the subject used a keyboard to type after the presentation of each sentence what he or she had heard. In the fMRI experiment, the subject indicated through a button press whether the intelligibility of the distorted sentences had increased at their second presentation. A block design was used in the fMRI experiment because of its higher detection power compared to that of event‐related designs (Liu, Frank, Wong, & Buxton, 2001; Maus, van Breukelen, Goebel, & Berger, 2012). We hypothesized that the brain activity during the first presentation of the distorted sentences (resulting in low intelligibility) is mainly restricted to auditory cortex, whereas during the second distorted presentation (resulting in high intelligibility) the high spatial resolution of fMRI reveals how the activity spreads also to the frontal and motor cortices, and to subcortical brain structures. Moreover, we expected to find intelligibility‐related modulations in brain activity between the first and second presentations of the distorted sentences in the vicinity of the auditory cortex, similarly as in E/MEG studies (Hakonen et al., 2016; Tiitinen et al., 2012).

## 2 METHODS

### 2.1 Subjects

Five subjects (three females, two males, mean age 30.4 years; standard deviation 7.2 years; range 23–40 years; all right‐handed) were tested in a behavioral experiment. A separate group of 20 subjects (10 females, 10 males, mean age 23.6 years, standard deviation 3.2; range 20–32 years; two left‐handed) participated in an fMRI experiment. The subjects were department staff members and university students, and all were native Finnish speakers. None of the subjects reported having hearing impairments or a history of neurological disorders. The project was approved by the Research Ethics Committee of Aalto University, and all subjects gave their written informed consent.

### 2.2 Stimulus material

To maximize the contrast of intelligibility between the first and the second presentations of the distorted sentences, the intelligibility of the distorted sentences when first presented should be minimized. Therefore, taking the set of 192 Finnish sentences from our previous study (Hakonen et al., 2016), we selected 150 sentences for the behavioral experiment and 108 sentences for the fMRI experiment that were the most difficult to understand in the distorted form. Thus, on the basis of the previous results, the mean intelligibility scores for the sentences used in the current behavioral and fMRI experiments were 22% and 16%, respectively (mean sentence duration 3.0 s, standard deviation 0.6 s, range: 1.7–4.6 s). These scores were calculated by scoring the stems and suffixes of the inflected words separately after correction of obvious spelling errors. The intact sentences were synthesized with a sample frequency of 44.1 kHz and an amplitude resolution of 16 bits. The distorted sentences were produced by resampling the intact sentences at 4.41 kHz, and compressing the resampled signals digitally through reduction in the amplitude resolution (bit rate) with the 1‐bit uniform scalar quantification (USQ) method (Gray, 1990; Liikkanen et al., 2007) where the temporal envelope of the signal is represented only by two levels of amplitude such that each signal sample is rounded off to its nearest amplitude level. In the following, we refer to the subsequent presentations of the sentences in the distorted, intact and, again, in distorted form as a “D‐I‐D” stimulus set.

### 2.3 Experimental design

#### 2.3.1 Behavioral experiment

In the behavioral measurements, the subject was presented with 15 D‐I‐D stimulus sets. Each set comprised one block of seven distorted sentences, followed by a block of five intact sentences (a subset of the previous seven), which was followed by the same seven distorted sentences as in the first block. The presentation order of the sentences was the same in each case (notwithstanding sentence omissions in the second block). Two of the sentences were only presented in the distorted form to investigate the effect of repetition on the intelligibility of the distorted sentences. Following the presentation of each sentence, the subject used a keypad to type what he/she had heard. The experiment began with a presentation of an additional stimulus set during which the subject was familiarized with the experiment. The experiment was carried out in a soundproofed listening booth, and the stimuli were delivered as a monophonic signal to the subject's ears through Sennheiser HD650 headphones. Sound intensity of the stimuli was set at 70 dB sound pressure level (SPL).

#### 2.3.2 fMRI experiment

**Figure 1:**
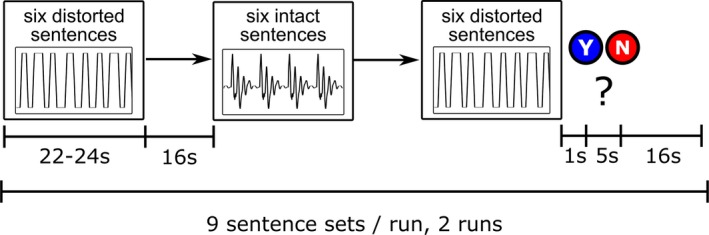
Setup of the fMRI experiment. The experiment was divided into two runs, each of which consisted of nine D–I–D stimulus sets. In each set, a block of six sentences was presented in the distorted, intact and, again, in the distorted form (unique set of sentences in each D–I–D set). The sentence blocks were separated by periods of 16 s without auditory stimulation. In a 1–6 s time window following the ending of the final block, the subject indicated with a button press (yes/no) whether the distorted sentences were more intelligible after the presentation of their intact counterparts

The fMRI experiment was divided into two 19‐min functional runs and one 6‐min anatomical run at the end of the scanning session. Each functional run consisted of 9 D‐I‐D stimulus sets, each of which comprised three blocks of six sentences (see Figure 1). The blocks were 22 or 24 s in duration. To prevent an overlap between the blood oxygenation level dependent (BOLD) responses elicited by each sentence block, the blocks were separated by periods of 16 s without auditory stimulation. Subjects were instructed to listen attentively to the sentences, to maintain their gaze on a central fixation cross, and to avoid moving during the duration of the experiment. After 1 s following the end of each D‐I‐D stimulus set, a question appeared on the screen for 5 s prompting the subject to indicate by a button press (yes/no) whether the distorted sentences were easier to understand when presented after the intact sentences. Half of the subjects responded with the right and the other half with the left hand. The stimuli were presented using Presentation software (Neurobehavioral Systems, http://www.neurobs.com/, RRID:SCR_002521), and the fixation cross and the visual prompt were projected to a mirror mounted on the head coil. The sentences were delivered as a monophonic signal to the subject's ears through MR‐compatible insertable earphones (Sensimetrics Corporation, Model S14, Malden, Massachusetts, USA, www.sens.com). Scan noise was attenuated by dense foam padding around the ears and head coil. Prior to the fMRI scanning, the subject was told that the auditory stimulation would include distorted and intact sentences, and a D‐I‐D stimulus set was presented to the subject on a computer screen to demonstrate the experiment. During a trial run before the experiment, the subject was presented with an intact and a distorted sentence during scanning, and the sound intensity of the sentences was adjusted to be both comfortable and loud enough to be heard over the scanner noise (the sound intensity averaged over subjects was 75 dB SPL).

### 2.4 Analysis of behavioral data

Intelligibility scores for the sentences were defined as the proportion of correct identifications and were computed by scoring the stems and suffixes of inflected words separately after obvious spelling errors had been corrected. Thereafter, for each of the 15 D‐I‐D sets and for each subject, the intelligibility scores were averaged separately over the first distorted sentence block, the intact sentence block, and the second distorted block. Intelligibility scores were calculated separately for the sentences that were presented only in the distorted form (i.e., 2 sentences per D‐I‐D stimulus set, 30 sentences in total) and for the sentences that were presented both in the distorted and intact forms (i.e., 5 sentences per D‐I‐D stimulus set, 75 sentences in total). The mean intelligibility scores were analyzed using a 15 × 2 × 2repeated‐measures analysis of variance (ANOVA) with the factors of stimulus set, sentence type (i.e., sentences presented only in the distorted form vs. sentences presented both in the distorted and intact forms) and ordinal position of the sentence (i.e., first vs. second presentation of the sentence). Post hoc comparisons (Newman–Keuls) were conducted when appropriate. The assumption of sphericity was tested by Mauchly's test. The intelligibility of the first and second blocks of the distorted sentences as well as of the intact sentence blocks were also assessed as a function of the ordinal position of the D–I–D stimulus set with linear mixed‐effect models while controlling for the impact of between‐subject variability both in speech intelligibility and in changes in speech intelligibility as a function of the ordinal position of the D–I–D stimulus set.

### 2.5 fMRI data acquisition

MR imaging was performed at the Advanced Magnetic Resonance Imaging Centre at Aalto University using a 3‐tesla MRI scanner (MAGNETOM Skyra, Siemens Healthcare, Erlangen, Germany) and a 32‐channel receiving head coil. Whole brain functional data were acquired with a T2*‐weighted echoplanar (EPI) sequence sensitive to the BOLD‐contrast (TR 2000 ms, echo time (TE) 30 ms, flip angle 76°, field of view (FOV) 220 mm, 3.4‐mm slice thickness, 37 oblique slices acquired in ascending order covering the whole brain in plane resolution). To achieve steady‐state magnetization, six dummy scans were performed at the beginning of each run. Anatomical images were acquired using a high‐resolution T1‐weighted Magnetization Prepared Rapid Gradient Echo (MPRAGE) pulse sequence (TR 2530 ms, TE 3.3 ms, flip angle 7°, 256×256 matrix, 176 sagittal slices, 1‐mm resolution). Head movements during scanning were minimized using padded cushions.

Heart rate and respiration signals time‐locked to the fMRI measurements were acquired using a BIOPAC MP150 Data Acquisition System (BIOPAC System, Inc.). For one half of the subjects, the pulse plethysmograph transducer (BIOPAC TSD200) was placed on the palmar surface of the subject's left index finger, and for the other half on the palmar surface of the subject's right index finger. Respiratory movements were measured using a respiratory‐effort transducer (BIOPAC TSD201) attached to an elastic respiratory belt, which was placed around the subject's chest. Both signals were sampled simultaneously at 1 kHz using RSP100C and PPG100C amplifiers for respiration and heart rate, respectively, and BIOPAC AcqKnowledge software (version 4.1.1).

### 2.6 fMRI analysis

#### 2.6.1 Preprocessing

Functional data were preprocessed with FSL (FMRIB Software Library v5.0, http://www.fmrib.ox.ac.uk/fsl/, RRID:SCR_002823; Smith, Jenkinson, & Woolrich, 2004; Woolrich et al., 2009). First, the images were converted into compressed Neuroimaging Informatics Technology Initiative (NIfTI‐1, http://nifti.nimh.nih.gov/dfwg, RRID:SCR_007117) format and reoriented to match the orientation of the Montreal Neurological Institute 152 (MNI 152) standard template in FSL using the fslreorient2std tool. Second, bias field was removed from the anatomical images using FMRIB's Automated Segmentation Tool (FAST; Zhang, Brady, & Smith, 2001), and nonbrain structures were removed from anatomical and functional images using Brain Extraction Tool (BET, https://fsl.fmrib.ox.ac.uk/fsl/fslwiki/BET; Smith, 2002). Thereafter, time‐series of fMRI volumes underwent slice time correction and motion correction using MCFLIRT (Jenkinson, Bannister, Brady, & Smith, 2002), and the first six dummy fMRI volumes were removed. Respiratory data was successfully recorded for 18 and cardiac data for 19 subjects. For these subjects, respiratory and cardiac artifacts were modeled and then removed from the fMRI data using the DRIFTER algorithm (Särkkä et al., 2012). Functional datasets were co‐registered to the subject's brain, extracted from T_1_‐weighted images, and these were then registered to the MNI152 standard space template with 2‐mm resolution. Both co‐registration steps were performed using FMRIB's Linear Image Registration tool (FLIRT, http://www.nitrc.org/projects/dwiregistration/, RRID:SCR_009461; Greve & Fischl, 2010; Jenkinson & Smith, 2001; Jenkinson et al., 2002) using nine degrees of freedom (translation, rotation, and scaling). The data was spatially smoothed using a Gaussian kernel with 10 mm full width at half maximum (FWHM).

#### 2.6.2 General linear model analysis

fMRI data was analyzed using a general linear model (GLM) as implemented in SPM12 (http://www.fil.ion.ucl.ac.uk/spm/, RRID:SCR_007037). We looked at brain activity related to the first presentation of the distorted sentences, to the presentation of the intact sentences, and to the second presentation of the distorted sentences. In each case, activity was modeled in each voxel using boxcar functions (square waves) convolved with a canonical hemodynamic response function. Both the data and the design matrices were high‐pass filtered at 256 s, and the resulting model was pre‐whitened by an autocorrelation AR(1) model. The following contrasts were calculated for each subject: (1) second distorted versus first distorted, (2) intact versus first distorted, and (3) intact versus second distorted. The first contrast between activity elicited by acoustically identical stimulation allowed us to identify brain areas reflecting speech intelligibility. The two other contrasts were used to study whether the activations related to intelligibility are specific to listening degraded speech signals or reflect speech comprehension more generally. For group analyses, the contrast images for each subject were submitted to a one‐sample *t*‐test. The resulting *t*‐maps were thresholded using nonparametric permutation tests using the SnPM toolbox (Nichols & Holmes, 2001; SnPM13, http://www.warwick.ac.uk/snpm, RRID:SCR_002092, 10,000 random permutations, cluster‐wise inference with a cluster‐forming threshold of *p* < .0001, cluster‐level results corrected for multiple comparisons using family‐wise error (FWE) at *p* < .05; the values were selected following the recommendations in the SnPM manual).

## 3 RESULTS

### 3.1 Behavioral results

**Figure 2:**
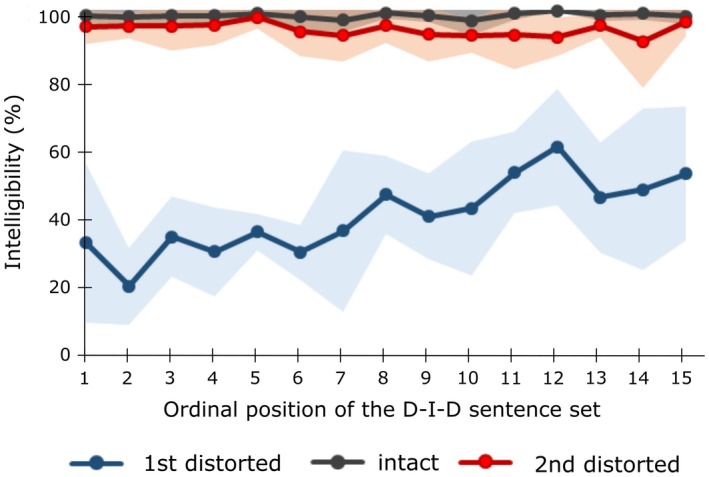
The mean intelligibility scores across the subjects for the sentences at their first presentation in the distorted form, at their presentation in the intact form, and at their second presentation in the distorted form. Shaded error bars indicate the standard error of the mean. Intelligibility increased after an exposure of their intact counterparts in each D–I–D stimulus set. Intelligibility of the distorted sentences upon their first presentation also increased as a function of the ordinal position of the stimulus set. The sentences presented only in the distorted form were excluded from the figure

The proportion of correct identifications of the distorted sentences averaged over the first presentations of all the stimulus sets was 42.8 ± 3.9%. At the second presentations, the distorted sentences were easier to understand, their average intelligibility being 73.7±2.8% [*F*(1,4) = 193.71, *p* < .001]. However, this increase in intelligibility depended on whether the intact version of the sentence (mean of the intelligibility scores of the intact sentences: 98.7 ± 0.7%) was present in the stimulus set [*F*(1,4) = 119.27, *p* < .001]. *Post hoc* comparisons revealed that the increase in intelligibility was stronger for the sentences that were also presented in the intact form (from 40.8 ± 4.9% to 94.6 ± 1.4%, *p *< .001) than for the sentences that were only presented in the distorted form (from 44.6 ± 4.0% to 52.9 ± 4.8%, *p* < .05). The assumption of sphericity was not violated in any of the analyses (Mauchley's test *p* = n.s.). Figure 2 shows the intelligibility scores for each D–I–D stimulus set. The intelligibility of the sentences at their first presentation in the distorted form increased as a function of the ordinal position of the D–I–D stimulus set [*F*(73) = 7.6, *p* < .001, *R* ^2^ = .56] but remained constant at the second presentation in the distorted form and at the presentation in the intact form.

### 3.2 fMRI Results

**Figure 3:**
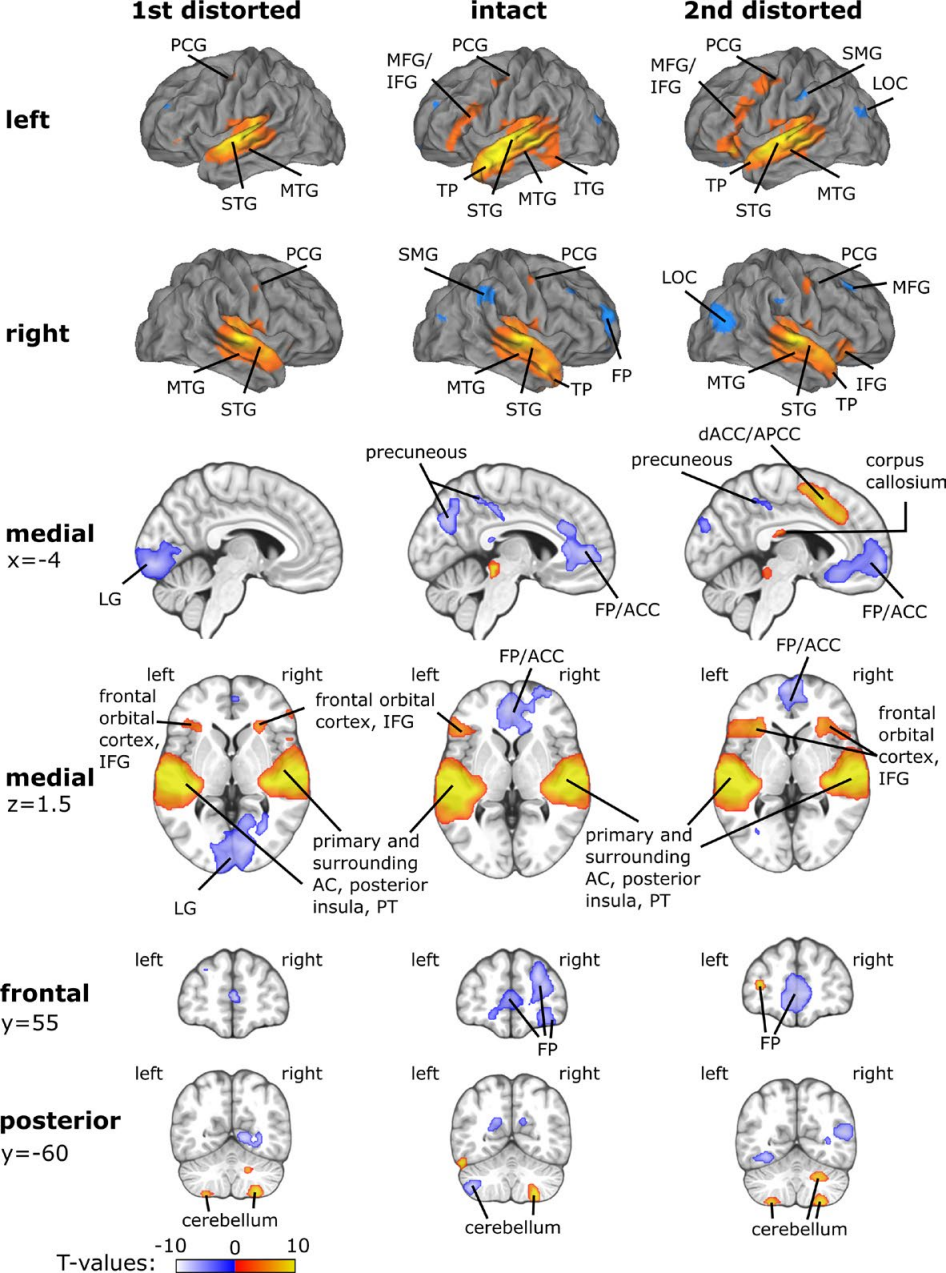
Blood oxygenation level dependent responses for the first presentations of the distorted sentences, the presentations of the intact sentences, and the second presentations of the distorted sentences. Activation maps were obtained using a cluster‐level nonparametric multiple comparisons procedure with a cluster‐forming threshold of *p* < .0001 and 10,000 random permutations. Cluster‐level results were family‐wise error‐corrected at *p* < .05

In the fMRI experiment, the subjects reported that the distorted sentences became more intelligible after the presentation of their intact counterparts for 17.1±1.3 of 18 D‐I‐D stimulus sets (range: 15–18). Figure 3 shows the fMRI activations during (1) the first presentation of the distorted sentences, (2) the presentation of the intact sentences, and (3) the second presentation of the distorted sentences, contrasted against the baseline without auditory stimulation (detailed results are given in supplemental Table S1; activation maps are in Neurovault: http://neurovault.org/collections/1626/). In all three cases, activity was found bilaterally in an area covering the primary and surrounding auditory cortex, the posterior insular cortex, the superior temporal gyrus (STG), the middle temporal gyrus (MTG), and the middle part of the precentral gyrus (PCG). The activity extended from the STG onto the posterior part of the inferior temporal gyrus (ITG) and to the temporal pole (TP) in the case of the intact sentences as well as to the TP in the case of the second presentation of the distorted sentences. Also, the first and second delivery of the distorted sentences both activated the left IFG. The dorsal anterior cingulate/paracingulate cortex (dACC/APCC) and the left frontal pole (FP) were activated by the second presentation of the distorted sentences. The presentations of the intact sentences and the second presentation of the distorted sentences resulted both in decreased activity within an area extending from the middle FP to the dACC/APCC. However, the deactivated area was larger at the second presentation of the distorted sentences. Additionally, the presentation of the intact sentences suppressed activity in the right‐hemispheric FP, the supramarginal gyrus (SMG) and the middle frontal gyrus, the left‐hemispheric posterior precuneous cortex, as well as bilaterally in the cerebellum, the occipitotemporal fusiform gyrus, and the lateral occipital cortex (LOC). At the first (low intelligibility) presentation of the distorted sentences, decreased activity was found in the lingual gyrus (LG), the left FP, and the left superior parietal lobe. The second presentation of the distorted sentences resulted in decreased BOLD responses in the right‐hemispheric LOC and middle frontal gyrus (MFG), the left‐hemispheric SMG and anterior precuneous cortex, as well as bilaterally in the LOC. In the left hemisphere, the deactivated area extended from the LG onto the occipitotemporal fusiform gyrus.

**Figure 4:**
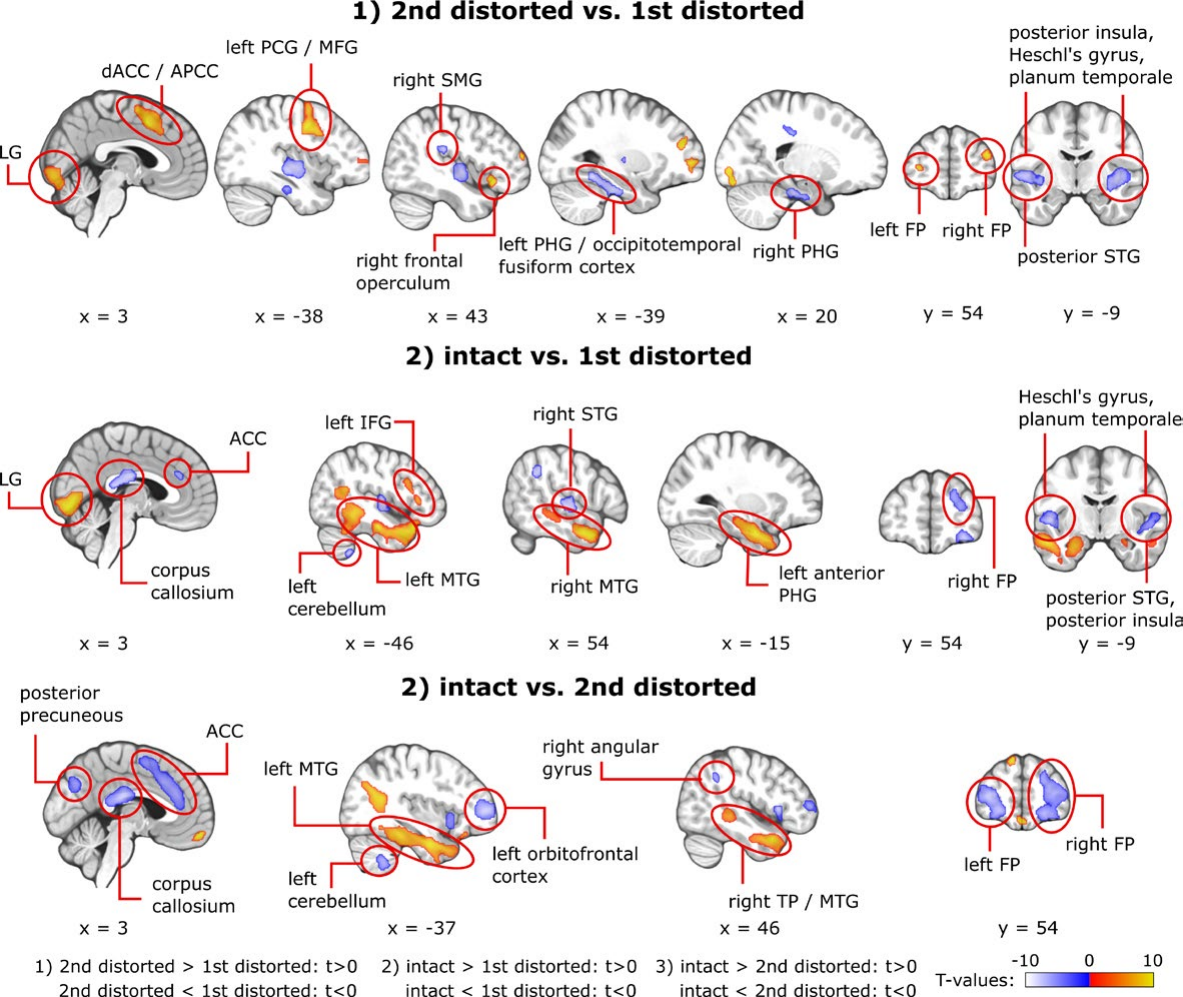
Significant blood oxygenation level dependent‐activations in the following contrasts: (1) the second (high intelligibility) presentation of the distorted sentences versus the first (low intelligibility) presentation of the distorted sentences, (2) the presentation of the intact sentences versus the first presentation of the distorted sentences, and (3) the presentation of the intact sentences versus the second presentation of the distorted sentences. Activation maps were obtained using a cluster‐level nonparametric multiple comparisons procedure with a cluster‐forming threshold of *p* < .0001, 10,000 random permutations and a cluster‐level family‐wise error correction at *p* < .05

As shown in Figure 4 (top, warm colors) and Table 1, there were six clusters where the second presentation of the distorted sentences lead to stronger BOLD‐activations than the first presentation. These clusters were situated in the LG, the dACC/APCC, the frontal operculum, and in the brain area extending from the left PCG to the left MFG. Moreover, two spherical clusters were found in the FP bilaterally. The dACC/APCC, the frontal operculum, and the brain area extending from the PCG to the left MFG became apparent in this contrast because they were activated at the second but not at the first presentation of the distorted sentences whereas LG became apparent because of its deactivation at the first presentation of the distorted sentences (see main effects in Figure 3). The left FP was deactivated at the first presentation and activated at the second presentation of the distorted sentences. The right FP became apparent only when contrasting the BOLD responses to the first and the second presentation of the distorted sentences but was not activated/deactivated in the main effects.

**Table 1:** Blood oxygenation level dependent‐activations for the contrasts (1) between the first and the second presentations of the distorted sentences, (2) between the presentation of the intact sentences and the first presentation of the distorted sentences and (3) between the presentation of the intact sentences and the second presentation of the distorted sentences

Brain region	*p*_FWE,cluster_	*k*	*T*‐value	MNI coordinates		
				x	y	z
*2nd distorted—1st distorted*						
Increased activity						
Paracingulate gyrus	0.0007	1055	7.90	2	10	48
Lingual gyrus	0.0004	1265	6.61	−4	−90	−12
Left precentral gyrus	0.0039	282	6.58	−36	0	40
Right frontal pole	0.0292	78	6.55	38	60	18
Right frontal orbital cortex	0.0057	223	5.90	38	24	–14
Left frontal pole	0.0303	76	5.70	−32	56	4
Decreased activity						
Right insular cortex	0.0008	762	6.93	38	−16	0
Right parietal operculum cortex	0.0104	174	6.28	46	−30	26
Left planum polare	0.0019	519	6.11	−52	−8	2
Right parahippocampal gyrus, posterior division	0.0115	161	5.65	20	−24	−26
Left temporal occipital fusiform cortex	0.0094	186	5.57	−30	−48	−12
*Intact—first distorted*						
Increased activity						
Left middle temporal gyrus, posterior division	0.0001	4528	10.36	−52	−12	−20
Right middle temporal gyrus, anterior division	0.0008	776	8.45	58	2	−24
Lingual gyrus	0.0002	1759	8.29	−6	−90	−8
Right middle temporal gyrus, posterior division	0.0027	435	7.58	40	−36	−8
Left angular gyrus	0.0035	389	7.14	−50	−52	16
Left inferior frontal gyrus, pars opercularis	0.0049	329	6.72	−52	16	26
Right parahippocampal gyrus, anterior division	0.0245	95	5.89	28	0	−30
Decreased activity						
Left Heschl's gyrus	0.0018	501	8.44	−52	−12	4
Right frontal pole	0.0083	197	8.16	32	52	−16
Corpus callosum	0.0013	625	7.80	8	−30	16
Right superior temporal gyrus, posterior division	0.0012	682	6.72	70	−22	8
Right frontal pole	0.0014	574	6.35	28	32	−8
Right frontal pole	0.0057	251	6.01	26	58	20
Right angular gyrus	0.0185	105	5.49	52	−46	34
Left cerebellum	0.0449	50	5.26	−44	−46	−52
Left planum temporale	0.0272	81	5.18	38	−30	14
Cingulate gyrus, anterior division	0.0292	75	5.11	0	32	26
*Intact—2nd distorted*						
Increased activity						
Left middle temporal gyrus, anterior division	0.0001	5930	12.68	−60	−6	−18
Right temporal pole	0.0002	2088	10.13	50	12	−26
Left frontal pole	0.0030	334	7.25	−10	54	42
Left angular gyrus	0.0007	827	7.11	−38	‐54	18
Right middle temporal gyrus, posterior division	0.0040	269	7.03	50	−34	−2
Frontal pole	0.0266	75	5.31	−2	58	−18
Decreased activity						
Right insular cortex	0.0001	5503	9.70	32	24	−2
Corpus callosum	0.0009	893	9.35	−4	–22	24
Left frontal pole	0.0006	1181	8.23	−32	50	6
Right precuneous cortex	0.0018	603	8.01	14	66	30
Left cerebellum	0.0059	235	7.07	−38	−52	−50
Left frontal orbital cortex	0.0047	283	6.64	−28	24	−6
Right angular gyrus	0.0439	58	5.49	48	−48	36

Results were obtained using a cluster‐level nonparametric multiple comparisons procedure based on permutation testing (cluster‐level results corrected for multiple comparisons using FEW correction at *p* < .05, a cluster‐forming threshold of *p* < .0001, 10,000 random permutations).

*p*_FWE,cluster_, *p*‐values, family‐wise error‐corrected at the cluster‐level; *k*, number of voxels in a cluster; *T*‐value, peak‐level *T*‐value; x, y, z (mm), coordinates in MNI space for each maximum peak‐level *T*‐value.

We found decreased activity for the second presentation of the distorted sentences in comparison to the first presentation bilaterally in the area extending from the posterior insular cortex to Heschl's gyrus (including Te1.2, Te1.0, and Te1.1) and planum temporale. Also, in the right hemisphere, the BOLD‐suppression peaked in the posterior insular cortex and, in the left hemisphere, it peaked in the planum polare. In the left hemisphere, the deactivated cluster extended from Heschl's gyrus onto the posterior STG. These brain areas were activated both at the first and the second presentations of the distorted sentences, and became apparent in the contrast because of the stronger activity at the first presentation of the distorted sentences. Additionally, for these contrasts, right‐hemispheric decreases of activity were found in the anterior SMG and the parahippocampal gyrus (PHG). In the left hemisphere, the activity decreased in the occipitotemporal fusiform gyrus. However, these areas were not activated/deactivated in the main effects.

In the MTG, the intact sentences elicited bilaterally stronger BOLD activity than the initially presented distorted sentences (Figure 4, middle; Table 1). In the left hemisphere, this increased activity spread from MTG to the TP, to the posterior part of ITG, and to PHG. In the right hemisphere, the activity enhancements were found in the middle and anterior MTG as well as in the TP. A cluster of increased activation was also found in the anterior part of the right PHG, but it was less pronounced than that in the left hemispheric PHG. Additionally, activity increased in the left IFG and in the LG. The areas where activity elicited by the intact sentences was lower than that elicited by the initially presented distorted sentences included the posterior corpus callosum, the ACC, three clusters in the right FP, the right angular gyrus, the left cerebellum, and, in both hemispheres, Heschl's gyrus (including Te 1.1, Te 1.0, and Te 1.2) and planum temporale. In the right hemisphere, this cluster of relative deactivation extended from Heschl's gyrus onto the posterior STG, to the posterior superior temporal sulcus (STS), and also to the posterior insula.

When contrasting responses to the intact sentences against responses to the second presentation of the distorted sentences, BOLD activity increased bilaterally in the TP, the MTG, and the LOC. This increase extended from the MTG and the TP onto the parahippocampal and fusiform gyri bilaterally (Figure 4, bottom; Table 1) and was more pronounced in the left hemisphere. Moreover, activity increased in the anterior superior frontal gyrus and in the middle orbitofrontal cortex. Activity decreases constituted clusters in the ACC/APCC, the posterior precuneous, the right angular gyrus, and the left cerebellum. Bilateral deactivations were found in the FP and the ventrolateral/anterior insular region of the IFG extending to the frontal operculum.

## 4 DISCUSSION

This study investigated the effects of previous matching experience on the comprehension of acoustically distorted speech. In the experiment, each subject was first presented with acoustically distorted sentences, then with the intact versions of the same set of sentences, and finally, with the distorted sentences again. We were particularly interested in whether the two acoustically identical instances of the distorted sentences were processed differentially in terms of behavioral and hemodynamic measures. The behavioral experiment demonstrated that the low intelligibility (mean: 41%) distorted sentences near to the perfect level (mean: 95%) after a single presentation of their intact counterparts. The first main finding of the fMRI experiment was *stronger* BOLD responses for the second compared to the first presentation of the distorted sentences in the bilateral dACC/APCC and FP, the right frontal opercular cortex, and in the area extending from the PCG to the MFG. The second main finding was *decreased* BOLD responses for the second compared to the first presentation of the distorted sentences in the right‐hemispheric PHG and SMG as well as in the area extending from the posterior insula to the posterior STG. These results suggest that both cortical activations and deactivations are associated with changes in the intelligibility of speech. This has implications for understanding how comprehension of noisy speech relies on memory‐based predictions and other executive functions, as detailed below.

### 4.1 Behavioral correlates for speech intelligibility

The distorted sentences were first difficult to understand, as evidenced by an average intelligibility of 41%. After hearing the intact versions of the sentences, the intelligibility of their distorted counterparts increased to the near‐perfect level of 95%. This effect was due to the subject hearing the intact versions of the sentences, rather than to the repetition of the distorted sentences, as is verified by the much smaller increase in intelligibility, from 45% to 53%, of the sentences that were presented only in the distorted form.

The 54‐percentage‐point increase in intelligibility for the distorted sentences after the presentation of their intact counterparts was stronger compared to our previous studies where the intelligibility increased 49% points (Tiitinen et al., 2012) and 34 percentage points (Hakonen et al., 2016). This could be due to two reasons. First, the sentences used in the current study were a subset of sentences, which in our previous investigation (Hakonen et al., 2016) proved to be particularly difficult to understand upon their first presentation in the distorted form. Second, the current study employed only six sentences per D–I–D set whereas the previous studies used 120 and 48, respectively. The relatively large increase in intelligibility with 120 sentences in Tiitinen et al. (2012) might be explained by the fact that the sentence set in this previous study was constructed from only seven starting words, three sentence stubs, and four ending words. Indeed, taken together, the above results imply that the memory system probed with the current paradigm might have a capacity limitation. Thus, it is possible that the accessibility of the memory trace of a particular sentence decays when the number of activated memory traces – and therefore also the number of competing predictions—increases due to the presentation of subsequent sentences (Tulving & Pearlstone, 1966). Another explanation would be that the memory trace decays when the time between the presentation of the intact sentences and the second presentation of the distorted sentences increases (Brown, 1958), and when enough time has elapsed from the presentation of the intact sentence, the memory trace may no longer be available. As a result, the distorted sentence may become unable to engage memory recall and the intelligibility may therefore remain at the low level.

Previous studies have found that presenting a disambiguating stimulus (i.e., text or intact speech) at the same time (Wild et al., 2012a) or immediately after (Clos et al., 2014; Davis et al., 2005; Hervais‐Adelman et al., 2012; Sohoglu et al., 2012) the presentation of distorted speech increases comprehension of distorted speech. The current results and those of our previous studies (Hakonen et al., 2016; Tiitinen et al., 2012) extend these findings by showing that improvements in comprehension last for at least tens of seconds. This implies that the disambiguating stimulus is represented in memory with a long decay time. Further, multiple memory representations of this kind can clearly be maintained concurrently. An interesting question for further research is how the time interval between the intact sentences and the following distorted sentences affects the intelligibility of the latter. That is, what is the lifetime of the memory trace imprinted by the intact sentence?

The intelligibility of the first presentation of the distorted sentences increased approximately 2% points for each consecutive D–I–D set. This gradual generalization of intelligibility reflects the ability of the human auditory system to adapt dynamically to degraded speech. The average intelligibility of the distorted sentences at their first presentation was higher in this study (41%) than in our previous study (16%). This might reflect differences in the subject populations. Also, in this study, the subject was presented with 15 consecutive D–I–D sets of six sentences whereas in the previous study the subject was presented with only a single D–I–D set of 48 sentences. Thus, it seems that consecutive presentations of the D–I–D stimulus sets enhance perceptual learning of distorted speech compared to the continual exposure to the distorted speech.

The increase in intelligibility may not have been identical in the behavioral and fMRI experiments. First, in the behavioral experiment, the encoding of the sentences involved both listening to them and then typing what was heard, whereas in the fMRI experiment, encoding was based only on the auditory presentation of the sentences. Therefore, the increase in intelligibility may have been stronger in the behavioral than in the fMRI experiment. Second, compared to the delays in the fMRI experiment, typing the sentences in the behavioral experiment increased the time between the presentations of the intact sentences and the second presentation of the distorted sentences. This, in contrast, may have resulted in increased intelligibility in the fMRI experiment. However, regardless of these possible differences between the two experiments, the subjects in the fMRI experiment indicated through a button press that in 94% of cases, the distorted sentences were more intelligible at the second than at the first presentation. Therefore, the associated differences in the brain responses are likely to reflect brain mechanisms underlying speech comprehension in acoustically suboptimal conditions.

### 4.2 Increased brain activity with speech intelligibility

The second (high intelligibility) presentation of the distorted sentences elicited more pronounced BOLD responses than the first (low intelligibility) one in an extended set of brain areas: bilaterally in the dACC/APPC and the FP, the right frontal operculum, as well as in the area extending from the left PCG to the left MFG. In these brain areas, there were no differences between the BOLD responses elicited by the intact sentences and those elicited by the first presentation of the distorted sentences. Thus, these brain areas are likely to be involved specifically in the comprehension of distorted speech rather than in speech comprehension in general. Interestingly, bilateral activations within the ACC, the FP, and in the frontal operculum have been associated with the retrieval mode in which incoming sensory information is treated as a “retrieval cue” for information stored in episodic memory (Lepage, Ghaffar, Nyberg, & Tulving, 2000; Tulving & Schacter, 1990). The retrieval mode has been shown to become activated in old‐new recognition judgments on whether an item has been previously presented (Lepage et al., 2000; Tulving et al., 1994). Thus, in view of our results, retrieval mode‐type processing might contribute to the processing of distorted sentences by treating these as retrieval cues that are compared to memory representations created during the processing of the intact counterparts of these sentences. If these cues are sufficient for triggering successful retrieval, this results in the comprehension of the sentence. According to this interpretation, the impact of memory in speech comprehension progressively increases when the quality of the acoustic signal decreases, until at the extreme forms of distortion, speech comprehension becomes a cued memory task. Related ideas have been presented in the Ease of Language Understanding (ELU) model that describes how and when working memory is involved in disambiguating acoustically distorted speech, and how it interacts with long‐term memory (LTM) during this process (Baddeley, 2000; Rönnberg, Rudner, Lunner, & Zekveld, 2010; Rönnberg et al., 2013; Rudner & Rönnberg, 2008). This model suggests that in easy listening conditions, sensory information matches with the corresponding syllabic phonological representation in semantic LTM, resulting in rapid memory retrieval. However, in suboptimal listening conditions, a mismatch between the distorted speech signal and the LTM trace engages an explicit memory retrieval mode where the incomplete sensory information is filled in with the information stored during previous experiences (i.e., during the presentation of the intact sentences in the case of this study). Further, indirect support for the frontal activations in our study signifying the involvement of memory retrieval in the processing of distorted speech comes from neuropsychological studies: prefrontal lesions tend to cause mild impairments in recognition memory, and this is likely to reflect the role the prefrontal cortex has in implementing goal‐directed processes which enhance memory formation, facilitate retrieval, and evaluate the appropriateness of retrieved information (for a review, see Ranganath & Knight, 2002). Our results would also agree with the current trend toward seeing memory as a predictive tool that allows rapid adaptation to the present and preparation for future situations; it has been proposed that this forward‐looking role, rather than the one which allows remembering past events, may be the core evolutionary significance of memory (Klein, 2013; Kveraga, Ghuman, & Moshe, 2007).

The increases in intelligibility and the concomitant changes in brain activity in our experiment may also represent a mixture of effects. In the behavioral experiment, the distorted sentences of the D–I–D set showed an increase in intelligibility of 54% points. However, there was an increase in 8% in intelligibility for those sentences repeated without the intervening intact counterpart, and the intelligibility of the first presentations increased during the session. Thus, while memory retrieval of the intact sentences was likely to be the main contributor to comprehension (as described above), perceptual learning seems to have been taking place might also be reflected in the BOLD responses. This should be addressed in future fMRI studies by including, for example, a control condition where the degraded sentences are repeated without the intervening intact sentences. Giraud et al. (2004) studied the effects of perceptual learning on brain activity by presenting subjects the same set of distorted sentences in two phases: before and after learning. This setup differs from ours in two important ways. First, the subjects in the study of Giraud et al. (2004) underwent perceptual training allowing them to decipher the distorted sentences in a generalized way. Second, the subjects were unaware during the first phase that the distorted sentences were speech signals and therefore were not expending effort to detect phonological cues. In the second phase of their study, the subjects realized that they were hearing distorted speech, and therefore the two phases differed not only in terms of speech comprehension but also in terms of phonological search and attention. The combined effect of these factors was reflected as an activation of the dACC/APCC, the MFG, and bilateral anterior insula. In contrast, the subjects in our study knew already from the start of the experiment that the signals were distorted speech signals, so it is unclear why phonological search and attention would have differed between our phases. However, the subjects were cued to expect intelligibility increases in the second block of distorted sentences, and it is therefore possible that listening was more effortful in these blocks. Thus, in view of the results of Giraud et al. (2004), it is possible that the stronger prefrontal and dACC/APCC activations in our experiment signaled not only increased comprehension but also successful phonological search. The dACC/APCC activation might also signify the engagement of an “executive” network which is activated in suboptimal listening conditions (Erb et al., 2013), and which signals the prefrontal cortex for a need of greater cognitive control in task situations where sensory information can be interpreted in several ways and hence could give rise to several, conflicting behavioral response patterns (Kerns et al., 2004; MacDonald, Cohen, Stenger, & Carter, 2000; Ridderinkhof, Ullsperger, Crone, & Nieuwenhuis, 2004). Our findings are also in line with results showing that the PCG becomes more activated when the intelligibility of vocoded speech increases as a result of pairing the vocoded stimulus with its original counterpart (Hervais‐Adelman et al.,^2012^) or as a result of perceptual learning, when the subject adapts to understand vocoded speech in the course of the presentations of vocoded sentences (Erb et al., 2013). Hervais‐Adelman et al. (2012) and Erb et al. (2013) proposed that PCG disambiguates distorted speech by comparing the degraded auditory signal with existing articulatory representations of speech sounds. In sum, increases in intelligibility in our experiment might have been supported by auditory search and further top‐down executive functions, as well as by the recruitment of articulatory representations of speech sounds.

### 4.3 Decreased brain activity with speech intelligibility

Compared to the BOLD activity elicited by the first presentation of the distorted sentences, activity decreased at the second presentation of the distorted sentences as well as at the presentation of the intact sentences within the brain area extending bilaterally from Heschl's gyrus to the middle STG and in the right hemisphere to the SMG. No differences were found between the BOLD responses to the second presentation of the distorted sentences and to the presentation of the intact sentences within these areas. Thus, BOLD responses even in the primary auditory cortex and surrounding areas do not simply reflect acoustic features of the signal but are also strongly modulated by previous experiences. The time span of these priming effects is 38–40 s, that is, the time lag between individual sentences in two consecutive stimulus blocks.

The above decrease in activity in temporal cortex seems to be contradicted by previous studies which found that BOLD responses *increase* bilaterally in temporal cortex when distorted speech is being disambiguated as a result of pairing it with its intact written or auditory counterparts (Clos et al., 2014; Hervais‐Adelman et al., 2012; Tuennerhoff & Noppeney, 2016) or as a result of perceptual learning (Giraud et al., 2004). These findings are in line with several studies that have associated temporal cortex with speech intelligibility (Davis & Johnsrude, 2007; Hickok & Poeppel, 2007; Narain et al., 2003). In contrast, our results showed BOLD decreases in temporal cortex between the first and second presentations of the distorted stimuli (see also Blank & Davis, 2016). This may suggest that activation of this area is not necessary for speech intelligibility in acoustically adverse conditions. In our study, the distorted sentences were presented 38–40 s after their intact counterparts whereas in previous studies, the distorted stimulus was presented simultaneously or immediately after the disambiguating stimulus. Thus, the previously reported activity increases may have reflected memory traces that decay much faster than 38–40 s. Indeed, the STG has been associated with phonological memory (Wong, Uppunda, Parrish, & Dhar, 2008) in which memory traces have a lifetime of only a few seconds (Baddeley, 2000).

Previous studies (Clos et al., 2014; Sohoglu & Davis, 2016; Sohoglu et al., 2012; Tuennerhoff & Noppeney, 2016) have suggested that predictive coding (Friston, 2005; Huang & Rao, 2011; Mumford, 1992; Rao & Ballard, 1999) underlies the instant increase in intelligibility of distorted speech signals when these are presented simultaneously with or immediately after the presentation of the disambiguating stimulus (e.g., a written or intact auditory counterpart of the speech stimulus). The predictive coding framework proposes that information residing in an internal predictive model is fed back from higher‐order cortical areas to lower‐level brain areas whose activity reflects the difference between auditory input and the predictive information, that is, the prediction error signal (Friston, 2005; Huang & Rao, 2011; Mumford, 1992; Rao & Ballard, 1999). This error signal is projected to the higher‐order cortical areas through feedforward connections to update the internal model. Applying this framework to our experimental paradigm, one would expect that the responses in the auditory (i.e., lower‐level) areas decrease at the second compared to the first presentation of the distorted sentences since the prediction error (i.e., the mismatch between the internal model and the auditory input) diminishes compared to the prediction error at the initial exposure of the distorted sentence when no predictive information is available. However, most of the previous fMRI studies that have used related experimental paradigms have not found the activity within the primary auditory cortex and surrounding areas to be modulated (Golestani, Hervais‐adelman, Obleser, & Scott, 2013; Hervais‐Adelman et al., 2012; Tuennerhoff & Noppeney, 2016; Zekveld et al., 2012; see also Wild et al., 2012a) when distorted speech of low initial intelligibility is rendered more intelligible by a prior or coincident presentation of a disambiguating stimulus. To explain these unexpected results, Wild et al. (2012a) and Tuennerhoff and Noppeney (2016) proposed that while the exposure to the disambiguating stimulus may decrease prediction error it may concurrently increase the precision of the prediction error, and that this, in turn, may be reflected as increased activity. Thus, these counteracting effects may cancel each other out and, as a result, no changes would be observed within the auditory cortices. In contrast, our results suggest that brain activity can, indeed, decrease in the auditory cortex and surrounding areas when a stimulus becomes disambiguated, similarly as observed in the visual cortex in a related study (Murray, Kersten, Olshausen, Schrater, & Woods, 2002). As an alternative explanation for the lack of modulation effects, the detection power of the event‐related designs of the previous studies may have been insufficient to reveal decreased activity. This interpretation would be in line with studies which, using a paradigm where the disambiguating speech stimulus is paired with the distorted stimulus, found EEG/MEG responses to decrease in the periauditory areas of the STG (Sohoglu & Davis, 2016; Sohoglu et al., 2012), that is, in an area partly overlapping with the area where activity decreased in the current study. As pointed out by the authors, the increased BOLD responses within the primary auditory cortex in the study of Wild et al. (2012a) may have been due to the subject paying more attention to the auditory sentence when it was presented with matching text.

Instead of reflecting the feedback from higher‐level brain areas, the BOLD‐reductions observed in this study could alternatively reflect locally originating modulations of neural activity (Grill‐Spector, Henson, & Martin, 2006; Henson, 2003). The decreased BOLD activity together with increased speech intelligibility may be explained, for example, with the sharpening model. This proposes that neurons coding word‐specific information send inhibitory feedback to the neurons coding features that are not essential for word identification, and that this results in a sparser and more specific neural representation of the word (Grill‐Spector et al., 2006; Henson, 2003; Wiggs & Martin, 1998). Further, these word‐specific memory representations might encode invariant global acoustic features of a word formulated as an average of the exposures to the various acoustic forms of that word during the subject's lifespan (Gagnepain et al., 2008). In this way, word‐specific memory templates could serve as a rapid adaptive filter that increases speech intelligibility in suboptimal listening conditions. While previous studies have linked decreased BOLD‐responses with shorter response times for making decisions about the stimuli (Gagnepain et al., 2008), our study suggests that in suboptimal conditions the behavioral benefit of the neural mechanisms underlying BOLD‐suppression might be increased speech intelligibility. Further analyses, for example using Dynamic Causal Modeling (Tuennerhoff & Noppeney, 2016), would be needed to establish whether the decreases in the activity in auditory cortex reflect bottom‐up (e.g., local sparse coding through representation sharpening) or top‐down (e.g., predictive coding) processing, or whether both mechanisms are involved. Further, it is improbable that activity changes alone are sufficient for deciding which information processing model is likely to be more correct. As pointed out by Blank and Davis (2016), both the predictive coding and sharpening models are consistent with the decreased BOLD responses these authors observed in the left posterior STS when distorted speech was disambiguated either by written text presented immediately before or by improving the speech signal acoustically. Evidence differentiating the models in favor of predictive coding was only found through analyzing the spatial multivoxel patterns in the STS.

## 5 CONCLUSIONS

Our results suggest that the intelligibility of degraded speech depends on the availability and accessibility of word‐specific memory representations that are rapidly created following exposure to intact speech and can then be swiftly activated. Specifically, single presentations of intact sentences increase considerably the intelligibility of their degraded counterparts, even when there is a long delay between the two. Whereas this dramatic increase in intelligibility was accompanied by enhanced BOLD responses in the prefrontal areas and in the dACC/APCC, a decrease in activity was observed bilaterally in the brain areas including the insular cortex, Heschl's gyrus, and the posterior STG, as well as in the right‐hemispheric SMG and PHG, and in the left‐hemispheric occipitotemporal fusiform gyrus. The activations in the prefrontal and cingulate cortices suggest the engagement of executive functions such as auditory search as well as the memory retrieval mode whereby the degraded sentences are treated as retrieval cues that are compared to information stored in memory. Therefore, the comprehension of degraded speech might rely on a process, which matches sensory information with corresponding memory representations. The reduced BOLD activity is consistent with predictive coding whereby responses in the sensory areas of cortex reflect prediction errors between incoming sensory information and internal models generated via previous experiences. Although a viable general explanation, this conclusion does not necessarily rule out the possibility where predictive information might be stored more locally, within the temporal brain areas, resulting in a more efficient processing of distorted speech.
