# Supplementary material for: Predictive processing increases intelligibility of acoustically distorted speech: Behavioral and neural correlates
Source: Brain Behav. 2017 Aug 4;7(9):e00789. doi: 10.1002/brb3.789 (PMC5607552; doi:10.1002/brb3.789)
Supplement: Supplementary file 1 [file BRB3-7-e00789-s001.docx]

**Table S1**: BOLD-activations for the first presentation of the distorted sentences, for the presentation of the intact sentences, and for the second presentation of the distorted sentences. Results are obtained using a cluster-level non-parametric multiple comparisons procedure based on permutation testing (a cluster-forming threshold of p < 0.0001, 10 000 random permutations, cluster-level results FEW-correcred at p < 0.05). $p_{FWE, cluster}$ = p-values, FWE-corrected at the cluster-level; k = number of voxels in a cluster; T-value = peak-level T-value; x, y, z (mm) = coordinates in MNI space for each maximum peak-level T-value.

| brain region | $\boldsymbol{p}_{\boldsymbol{FWE,Cluster}}$ | k | T-value | MNI coordinates | | |
| --- | --- | --- | --- | --- | --- | --- |
| **1st distorted presentation** |  |  |  | **x** | **y** | **z** |
| ***increased*** |  |  |  |  |  |  |
| Left Heschl’s gyrus | 0.0001 | 6433 | 18.21 | – 54 | – 16 | 2 |
| Right superior temporal gyrus | 0.0001 | 6601 | 14.94 | 62 | – 14 | 0 |
| Right cerebellum | 0.0048 | 249 | 7.32 | 26 | – 62 | – 60 |
| Right frontal orbital cortex | 0.0066 | 191 | 6.33 | 26 | 28 | 0 |
| Left cerebellum | 0.0387 | 58 | 6.21 | – 26 | –62 | – 62 |
| Left precentral gyrus | 0.0076 | 176 | 5.50 | – 50 | – 6 | 56 |
| Left inferior frontal gyrus, pars triangularis | 0.0072 | 181 | 5.43 | – 54 | 36 | – 4 |
| Left inferior frontal gyrus, pars opercularis | 0.0377 | 59 | 5.34 | – 44 | 12 | 18 |
| Right precentral gyrus | 0.0198 | 98 | 5.33 | 52 | – 6 | 44 |
| Right cerebellum | 0.0483 | 47 | 5.15 | 18 | – 58 | – 34 |
| ***decreased*** |  |  |  |  |  |  |
| Lingual gyrus | 0.0001 | 3323 | 9.20 | –4 | –90 | –8 |
| Left frontal pole | 0.0095 | 214 | 7.15 | –36 | ­44 | 28 |
| Left superior parietal lobe | 0.0893 | 49 | 5.89 | –28 | –50 | 40 |
| Right frontal pole | 0.0481 | 78 | 5.46 | 16 | 62 | 26 |
| Paracingulate gyrus | 0.0775 | 55 | 5.02 | 2 | 54 | 8 |
| **intact presentation** |  |  |  |  |  |  |
| ***increased*** |  |  |  |  |  |  |
| Left planum temporale | 0.0001 | 14641 | 19.43 | – 50 | – 20 | 0 |
| Right superior temporal gyrus | 0.0001 | 8032 | 15.42 | 62 | – 12 | 0 |
| Right cerebellum | 0.0075 | 203 | 7.14 | 24 | – 62 | – 52 |
| Parahippocampal gyrus | 0.0131 | 128 | 6.62 | 16 | – 26 | –10 |
| Right cerebellum | 0.0044 | 319 | 6.05 | 18 | – 78 | –46 |
| Left precentral gyrus | 0.0020 | 583 | 5.96 | -48 | – 2 | 54 |
| Right precentral gyrus | 0.0074 | 207 | 5.61 | 52 | – 8 | 50 |
| Left putamen | 0.0434 | 53 | 5.44 | -22 | 0 | 10 |
| ***decreased*** |  |  |  |  |  |  |
| Left precuneous cortex | 0.0013 | 1103 | 8.78 | –16 | –70 | 20 |
| Left cerebellum | 0.0013 | 947 | 8.74 | –42 | –64 | –48 |
| Right frontal pole | 0.0003 | 4192 | 8.71 | 26 | 60 | 22 |
| Right supramarginal gyrus, posterior division | 0.0022 | 615 | 7.57 | 52 | –44 | 36 |
| Precuneous cortex | 0.0017 | 785 | 7.17 | 4 | –40 | 46 |
| Right cerebellum | 0.0087 | 173 | 6.91 | 38 | –42 | –48 |
| Left lateral occipital cortex | 0.0121 | 132 | 6.20 | –36 | –86 | 18 |
| Right middle frontal gyrus | 0.0061 | 229 | 5.93 | 42 | 26 | 44 |
| Right temporal occipital fusiform cortex | 0.0120 | 133 | 5.68 | 32 | –46 | –10 |
| Left temporal occipital fusiform cortex | 0.0372 | 60 | 5.58 | –28 | –46 | –10 |
| Right lateral occipital cortex, superior division | 0.0183 | 102 | 5.53 | 38 | –76 | 18 |
| **2nd distorted presentation** |  |  |  |  |  |  |
| ***increased*** |  |  |  |  |  |  |
| Left Heschl’s gyrus | 0.0001 | 10416 | 16.72 | – 54 | – 16 | 2 |
| Right superior temporal gyrus | 0.0001 | 7649 | 15.77 | 64 | – 14 | 0 |
| Paracingulate gyrus | 0.0005 | 1368 | 8.45 | – 2 | 12 | 54 |
| Right cerebellum | 0.0036 | 247 | 7.92 | 26 | – 64 | – 60 |
| Left cerebellum | 0.0133 | 114 | 7.78 | – 26 | – 62 | –62 |
| Corpus callosum | 0.0072 | 159 | 7.55 | 6 | – 26 | 20 |
| Right cerebellum | 0.0010 | 538 | 6.88 | 16 | – 78 | -40 |
| Right precentral gyrus | 0.0018 | 372 | 6.80 | 52 | – 8 | 46 |
| Right inferior frontal gyrus, pars opercularis | 0.0169 | 99 | 6.54 | 40 | 14 | 24 |
| ***decreased*** |  |  |  |  |  |  |
| Left lateral occipital cortex | 0.0009 | 768 | 8.57 | -28 | -70 | 10 |
| Middle frontal pole | 0.0002 | 2322 | 8.35 | -6 | 62 | 4 |
| Right lateral occipital cortex, superior division | 0.0002 | 1675 | 8.32 | 52 | -64 | 18 |
| Left supramarginal gyrus, anterior division | 0.0073 | 197 | 7.46 | -58 | -30 | 36 |
| Right temporal occipital fusiform cortex | 0.0009 | 886 | 7.33 | 36 | -44 | -12 |
| Left precuneous cortex | 0.0060 | 232 | 7.00 | -12 | -38 | 46 |
| Right middle frontal gyrus | 0.0034 | 352 | 6.64 | 26 | 28 | 46 |
| Left lateral occipital cortex | 0.0132 | 634 | 6.62 | -32 | -56 | -12 |
